# Aspergillus-Specific IgG Antibodies are Associated With Fungal-Related Complications and Chronic Lung Allograft Dysfunction After Lung Transplantation

**DOI:** 10.3389/ti.2023.10768

**Published:** 2023-02-16

**Authors:** Hanne Beeckmans, Elfri Van Roy, Janne Kaes, Annelore Sacreas, Vincent Geudens, Astrid Vermaut, Lynn Willems, Xin Jin, Saskia Bos, Arno Vanstapel, Jan Van Slambrouck, Michaela Orlitova, Bart Vanaudenaerde, Laurens J. Ceulemans, Dirk Van Raemdonck, Arne P. Neyrinck, Laurent Godinas, Lieven J. Dupont, Geert M. Verleden, Robin Vos

**Affiliations:** ^1^ Laboratory for Respiratory Diseases and Thoracic Surgery, Department of Chronic Diseases, Metabolism and Ageing, Faculty of Medicine, KU Leuven, Leuven, Belgium; ^2^ Department of General Internal Medicine, University Hospitals Leuven, Leuven, Belgium; ^3^ Department of Development and Regeneration, Faculty of Medicine, KU Leuven, Leuven, Belgium; ^4^ Translational and Clinical Research Institute, Faculty of Medical Sciences, Newcastle University, Newcastle upon Tyne, United Kingdom; ^5^ Department of Thoracic Surgery, University Hospitals Leuven, Leuven, Belgium; ^6^ Department of Anesthesiology, University Hospitals Leuven, Leuven, Belgium; ^7^ Department of Respiratory diseases, University Hospitals Leuven, Leuven, Belgium

**Keywords:** lung transplantation, chronic lung allograft dysfunction, fungal infection, IgG, IgE, *Aspergillus*, molds

## Abstract

Fungal exposure and sensitization negatively affect outcomes in various respiratory diseases, however, the effect of fungal sensitization in lung transplant (LTx) recipients is still unknown. We performed a retrospective cohort study of prospectively collected data on circulating fungal specific IgG/IgE antibodies, and their correlation with fungal isolation, chronic lung allograft dysfunction (CLAD) and overall survival after LTx. 311 patients transplanted between 2014 and 2019 were included. Patients with elevated *Aspergillus fumigatus* or *Aspergillus flavus* IgG (10%) had more mold and *Aspergillus* species isolation (*p* = 0.0068 and *p* = 0.0047). *Aspergillus fumigatus* IgG was specifically associated with *Aspergillus fumigatus* isolation in the previous or consecutive year (AUC 0.60, *p* = 0.004 and AUC 0.63, *p* = 0.022, respectively). Elevated *Aspergillus fumigatus* or *Aspergillus flavus* IgG was associated with CLAD (*p* = 0.0355), but not with death. *Aspergillus fumigatus*, *Aspergillus flavus* or *Aspergillus niger* IgE was elevated in 19.3% of patients, but not associated with fungal isolation, CLAD or death. Mold isolation and *Aspergillus* species isolation from respiratory cultures were associated with CLAD occurrence (*p* = 0.0011 and *p* = 0.0005, respectively), and *Aspergillus* species isolation was also associated with impaired survival (*p* = 0.0424). Fungus-specific IgG could be useful in long-term follow-up post-LTx, as a non-invasive marker for fungal exposure, and thus a diagnostic tool for identifying patients at risk for fungal-related complications and CLAD.

## Graphical Abstract

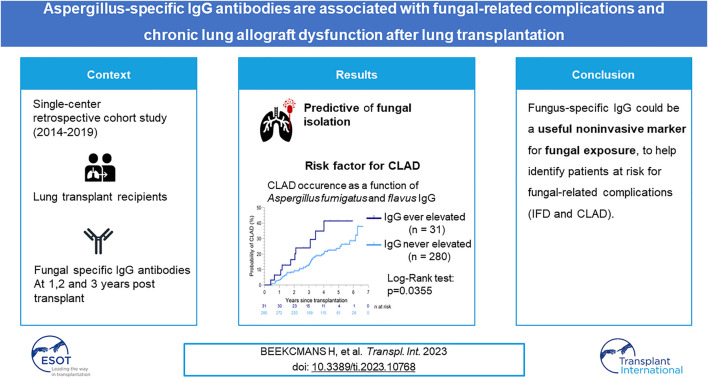

## Introduction

Fungal infections are prevalent after lung transplantation (LTx), where they are observed in 15%–35% of all patients. These numbers are notably higher than after other solid-organ transplantations (1). *Aspergillus fumigatus* is a known risk factor for chronic lung allograft dysfunction (CLAD), the leading cause of death beyond the first year after LTx, even in patients that are only colonized without signs of infection (2–5). However, respiratory samples are not always readily available, as not all patients produce sputum. Subclinical presence of *Aspergillus fumigatus* could thus remain undetected. The role of other *Aspergillus* species or non-*Aspergillus* molds in the pathogenesis of CLAD is currently unclear (2, 3, 5).

Serum *Aspergillus* IgG (or precipitins) and *Aspergillus* IgE are currently mostly used in the diagnosis of chronic pulmonary aspergillosis and allergic bronchopulmonary aspergillosis (ABPA) (6, 7). However, the value of fungal-specific IgG or IgE in the absence of clinical symptoms is less clear. Fungal sensitization is defined as an immune-mediated response to a fungus without signs of inflammation or tissue damage, as opposed to ABPA (8). Recent studies in other chronic respiratory diseases like asthma, chronic obstructive lung disease, and cystic fibrosis (CF) have suggested an adverse effect of fungal sensitization, even without signs of infection (8–13). Particularly in asthma, the importance of fungal sensitization on respiratory disease control is well documented, and it is generally accepted that exposure to allergens, like fungal proteins, can trigger and exacerbate asthma symptoms (8–11).

Fungal sensitization can occur through a normal and an allergic pathway. “Normal” fungal sensitization occurs through activation of the immune system, where TH1- and TH17-cells help to build protective immunity against fungal pathogens *via* release of cytokines, which then stimulate phagocytes as well as B-cells to produce specific IgG antibodies (delayed response). On the other hand, patients may experience allergic fungal sensitization, in which exposure to fungal allergens causes an allergic reaction *via* a type 2 immune response, with activation of TH2-cells in regional lymph nodes. These immune cells secrete cytokines and stimulate B-cells to produce specific antifungal IgE-antibodies (8). Allergic fungal sensitization thus is the result of immune reactions in the airways, leading to hyperinflammation, causing deterioration of respiratory symptoms. As all LTx recipients are immunosuppressed to avoid graft rejection and therefore have a decreased immune activity, occurrence of fungal sensitization in transplanted patients could mean that their immune system might not be as suppressed as one would expect. Allergic fungal sensitization may be a sign of attenuated immunosuppression, possibly contributing to later onset of chronic rejection. Detection of fungus-specific IgG antibodies on the other hand, may reflect past fungal presence in the airways (10), and could thus potentially be used as a measure for fungal exposure, independent of airway sampling, identifying patients at a higher risk of (invasive) fungal infections and CLAD.

In this retrospective study, we therefore aimed to explore the prevalence of fungal sensitization after LTx, and its possible effects on occurrence of (invasive) fungal infections, CLAD and survival.

## Materials and Methods

### Study Population

Patients who underwent LTx at UZ Leuven between September 2014 and December 2019 were evaluated for inclusion. Inclusion criteria were: 1) survival to 1 year post-LTx and 2) availability of ≥1 blood sample for specific IgG/IgE antibodies to *Aspergillus fumigatus*, *Aspergillus flavus* and specific IgE antibodies to Aspergillus niger, obtained during annual check-up at 1, 2 or 3 years post-LTx. Data regarding fungal isolation from respiratory samples, clinical phenotype of the fungus, CLAD onset and survival were obtained from the electronic patient files. The study was approved by the local Ethics Committee (University Hospitals Leuven, Belgium—S63978), and all patients provided written informed consent.

### Data Collection: Blood Samples

Blood samples with IgG/IgE measurement were collected during the pre-transplant work-up prior to listing for transplant, and at every annual post-transplant check-up, taking place between September 2017 and January 2021. In these samples total protein level, specific IgG’s, total IgE, and specific IgE’s were measured using ImmunoCAP fluoroenzyme immunoassay, with a Phadia™ 250 instrument for IgG measurements and a Phadia™ 1000 instrument for IgE measurements (ThermoFisher, Waltham, Massachusetts, United States), as per institutional standard operating procedures.

Specific IgG’s against *Aspergillus fumigatus* (Gm3) and *Aspergillus flavus* (Gm228) were determined. For the specific IgE’s, the following were assessed: *Aspergillus fumigatus* (m3), *Aspergillus flavus* (m228) and *Aspergillus niger* (m207).

For specific IgG’s the lower limit of detection was 2.0 mg/L, values >50.0 mg/L for *Aspergillus fumigatus* and *Aspergillus flavus* were considered positive per assay protocol. The lower limit of detection for the specific IgE’s was 0.10 kU/L, and every detectable value was considered positive.

### Data Collection: Airway Samples

Airway samples were collected at fixed post-transplant check-ups on day 1, 30, 90, 180, 365, 540, and 720. They were also additionally obtained when patients exhibited respiratory symptoms with or without a fall in FEV1, or presented with an abnormal chest x-ray or computed tomography during routine post-LTx follow-up.

Respiratory specimens, acquired *via* sputum or bronchoalveolar lavage, were cultured using Sabouraud dextrose agar and CHROMagar *Candida* growth mediums per institutional protocol, and considered positive if fungi were detected, with subsequent species-identification.

Positive fungal cultures were further categorized as *Aspergillus* species (*Aspergillus fumigatus*, *Aspergillus flavus*, *Aspergillus niger*) or molds (exclusion of yeasts, included molds in our cohort were: *Aspergillus fumigatus*, *Aspergillus flavus*, *Aspergillus niger*, *F. fomentarius*, *Fusarium* species, *P. nariotii*, *Penicillium* species, *Polyporales* species, *P. lilacinum*, *R. argilacea*, *R. microsporus*, *S. apiospermum*, *Scopulariopsis* species, *Talaromyces* species).

For further analysis regarding CLAD and survival, fungal cultures in the first postoperative month were excluded, as these were expected to be confounded by flora from the donor lung.

### CLAD Definition

CLAD was defined according to the latest ISHLT consensus paper (14). Bronchiolitis obliterans syndrome (BOS) was defined as a FEV1 decline of ≥20% with an obstructive PFT pattern, in absence of persistent radiologic opacities or TLC decline. Restrictive allograft syndrome (RAS) was defined as a FEV1 decline of ≥20% accompanied with a restrictive PFT pattern (TLC decline of ≥10% compared to baseline) and persistent opacities on chest x-ray or computed tomography (CT).

### Antifungal Prophylaxis and Treatment

All patients received standard prophylaxis with nebulized amphotericin B lipid complex for 1 month after LTx (patients with bronchial anastomosis necrosis at 1 month after LTx received prolonged targeted prophylaxis up to 3 months after LTx). No systemic antifungal prophylaxis with azoles was given in our cohort. Antifungal treatment with systemic voriconazole, posaconazole, isavuconazole, or amphotericin B was initiated at the treating clinician’s decision, in case of fungal disease.

### Fungal Disease

Clinical phenotyping (invasive fungal disease (IFD) or non-IFD) of fungi isolated from cultures was performed according to the latest EORTC-MSGERC and ISHLT consensus paper (15, 16). Patients who fulfilled ISHTL criteria for fungal pneumonia, tracheobronchitis or anastomotic infection were deemed probable or proven IFD according to EORTC-MSGERC criteria (depending on histological criteria). Patients who had clinical symptoms without radiological or endobronchial criteria, as well as patients who fulfilled ISHLT criteria for fungal colonization (i.e., no clinical symptoms and no radiological or endobronchial criteria) were categorized as non-IFD fungal isolation.

### Statistics

Outcome analysis was performed on 1st June 2021 (6 months after the last measurement of IgE/IgG). Calculations were performed in GraphPad Prism 9.3.1. and R. Normality was tested by the Shapiro–Wilk test; none of the continuous variables were normally distributed. Friedman test was used to evaluate IgG levels over time, and Spearman correlation to evaluate the relationship between IgG pre- and post-LTx. Log-rank test and receiver operating characteristic analysis were used to compare mortality, occurrence of CLAD and occurrence of fungal isolation. Univariate comparisons between groups were performed by Wilcoxon rank-sum test and presented as median and interquartile range. For comparison of discrete variables, Chi-squared test and Fisher’s exact test were used when appropriate, and presented as absolute numbers and percentages. A *p*-value <0.05 was considered significant.

## Results

### Patient Demographics

**FIGURE 1:**
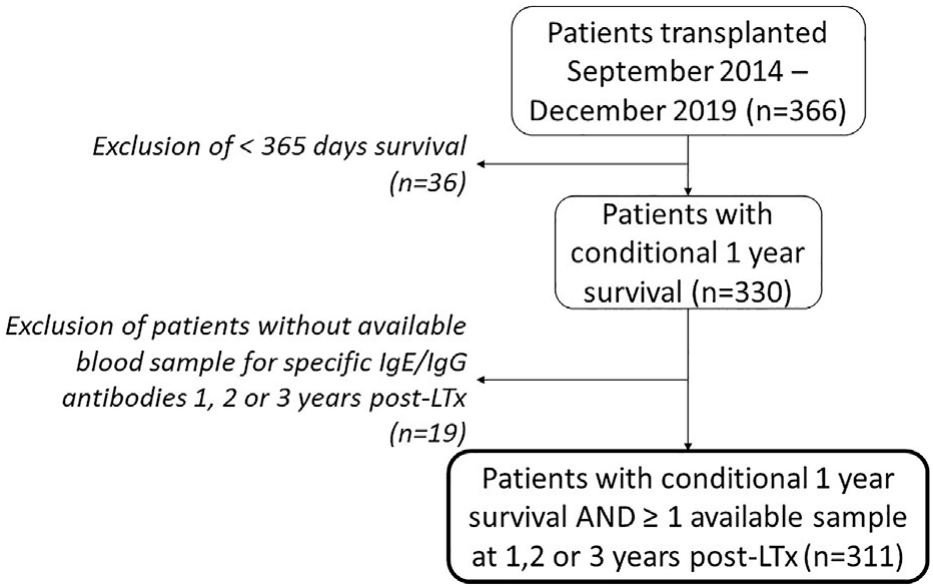
Patient inclusion flowchart. Abbreviations: LTx, lung transplantation.

Of the 366 consecutive patients who underwent LTx at our center in the aforementioned time interval, a total of 311 patients were included (Figure 1), of which *Aspergillus fumigatus* IgG/IgE antibody data were available in 201 (64%), 192 (62%) and 191 (61%) patients at respectively 1, 2 or 3 years post-LTx. The number of available samples per patient was 1 in 119 (38.3%) patients, 2 in 107 (34.4%) patients and 3 in 85 (27.3%) patients. Patient characteristics are listed in Table 1. IgG/IgE blood samples are presented in Table 2.

**TABLE 1:** Patient characteristics.

Male gender, n (%)	154 (49.5%)
Age, median [IQR] (years)	58 [46–62]
Underlying lung disease	
Chronic obstructive pulmonary disease, n (%)	165 (53.0%)
Interstitial lung disease, n (%)	56 (18.0%)
CF and non-CF bronchiectasis, n (%)	55 (17.7%)
Pulmonary hypertension, n (%)	7 (2.3%)
Other, n (%)	28 (9.0%)
Type of LTx	
Bilateral SSL, n (%)	300 (96.5%)
Combined liver-lungs, n (%)	7 (2.3%)
Combined heart-lungs, n (%)	3 (1.0%)
Combined kidney-lungs, n (%)	1 (0.3%)
CLAD diagnosis, n (%)	67 (21.5%)
Type BOS, n (%)	48 (71.6%)
Type RAS, n (%)	19 (28.4%)
BOS to RAS phenotype switch, n (%)	4 (6.0%)
Available blood samples per patient, median	2
Patients with 1 sample, n (%)	119 (38.3%)
Patients with 2 samples, n (%)	107 (34.4%)
Patients with 3 samples, n (%)	85 (27.3%)

CF, cystic fibrosis; SSL, sequential single-lung; CLAD, chronic lung allograft dysfunction; BOS, bronchiolitis obliterans syndrome; RAS, restrictive allograft syndrome.

**TABLE 2:** IgG and IgE levels.

Available samples, n	Pre-LTx	Year 1 post-LTx	Year 2 post-LTx	Year 3 post-LTx
	308	201	192	192
Samples with positive IgG values				
A. fumigatus, n (%)	98 (31.8%)	18 (9.0%)	13 (6.8%)	13 (6.8%)
Median value in positive samples (IQR), mg/L	74.75 (58.18–112.80)	63.95 (55.78–82.75)	67.4 (53.10–78.75)	84.7 (61.70–123.0)
A. flavus, n (%)	NA	6 (3.0%)	9 (4.7%)	10 (5.2%)
Median value in positive samples (IQR), mg/L		69.5 (63.43–94.0)	63.7 (58.40–71.75)	78.9 (63.08–113.3)
Samples with positive IgE values				
A. fumigatus, n (%)	NA	29 (14.4%)	31 (16.1%)	40 (20.8%)
Median value in positive samples (IQR), mg/L		0.6 (0.27–2.09)	1.22 (0.36–3.10)	0.48 (0.20–2.29)
A. flavus, n (%)	NA	5 (2.5%)	12 (6.3%)	10 (5.2%)
Median value in positive samples (IQR), mg/L		0.58 (0.19–9.97)	0.2 (0.14–0.67)	0.53 (0.15–2.10)
*A. niger*, n (%)	NA	8 (4.0%)	9 (4.7%)	8 (4.2%)
Median value in positive samples (IQR), mg/L		0.26 (0.12–0.73)	0.18 (0.14–1.10)	1.28 (0.28–2.40)

LTx, lung transplantation; A., *Aspergillus;* NA, not available.

### Fungal IgG/IgE—Fungal Isolation

Elevated *Aspergillus fumigatus* or *Aspergillus flavus* IgG at 1, 2 or 3 years post-LTx was detected in 31/311 (10%) patients. Characteristics of patients with or without elevated Aspergillus species IgG are depicted in Table 3. Detectable *Aspergillus fumigatus* IgG levels at 1, 2 or 3 years post-LTx were not significantly different, but there was a clear reduction in *Aspergillus fumigatus* IgG levels pre-vs. post-LTx (*p* < 0.0001). There was a correlation between *Aspergillus fumigatus* IgG levels pre- and post-LTx (r_s_ = 0.6055; *p* < 0.0001).

**TABLE 3:** Patient characteristics in patients with ever vs. never elevated *Aspergillus fumigatus* or *flavus* IgG after LTx.

	*A. fumigatus* or *A. flavus* IgG ever elevated at 1, 2 or 3 years post-LTx	*A. fumigatus* or *A. flavus* IgG never elevated at 1, 2 and 3 years post-LTx	*p*-value
Patients (%)	31 (9.97%)	280 (90.03%)	
Men (%)	15 (48.3%)	139 (49.6%)	
Age (years)	58 (44–61)	58 (46–62)	0.9611
LTx indication (%)			0.1261
BRECT	1 (3.2%)	5 (1.8%)	
CLAD	2 (6.5%)	12 (4.3%)	
CF	8 (25.8%)	44 (15.7%)	
Emphysema	17 (54.8%)	149 (53.2%)	
Pulmonary GVHD	1 (3.2%)	3 (1.1%)	
ILD	1 (3.2%)	54 (19.3%)	
PH	0 (0%)	5 (1.8%)	
Other	1 (3.2%)	8 (2.9%)	
Clinical phenotype (%)			**0.0282**
BOS	4 (12.9%)	41 (14.6%)	
RAS	4 (12.9%)	15 (5.4%)	
BOS to RAS	2 (6.5%)	2 (0.7%)	
Stable	21 (67.7%)	223 (79.6%)	
Ever mold isolation after POD 30 (%)	21 (67.7%)	115 (41.1%)	**0.0068**
Ever *Aspergillus* species isolation after POD 30 (%)	18 (58.1%)	88 (31.4%)	**0.0047**
Deceased (%)	4 (12.9%)	22 (7.9%)	0.3096

LTx, lung transplantation; A., *Aspergillus*; BRECT, bronchiectasis; CLAD, chronic lung allograft dysfunction; CF, cystic fibrosis; GVHD, graft-versus-host-disease; ILD, interstitial lung disease; PH, pulmonary hypertension; BOS, bronchiolitis obliterans syndrome; RAS, restrictive allograft syndrome; POD, postoperative day.

Bold represent significant *p*-values.

Elevated *Aspergillus fumigatus* IgG was associated with *Aspergillus fumigatus* isolation from respiratory samples in the following year (AUC 0.63, *p* = 0.022) and the previous year (AUC 0.60, *p* = 0.004). Elevated *Aspergillus flavus* IgG was not significantly associated with *Aspergillus flavus* isolation in the previous or consecutive year (although our study was probably underpowered for this analysis, with only 16 patients with elevated *Aspergillus flavus* IgG, and only 12 isolations from respiratory cultures). During the overall study period (up till 6 months after last IgG measurement) patients that ever had an increased *Aspergillus fumigatus* or *flavus* IgG demonstrated more mold and *Aspergillus* species isolation (*p* = 0.0068 and *p* = 0.0047, respectively, Table 3).

Elevated *Aspergillus fumigatus, flavus,* or *niger* IgE at 1, 2 or 3 years post-LTx was detected in 60/311 (19.3%) of patients, but was not associated with occurrence of *Aspergillus* species isolation in the consecutive or previous year.

### Fungal IgG/IgE—CLAD/Survival

**FIGURE 2:**
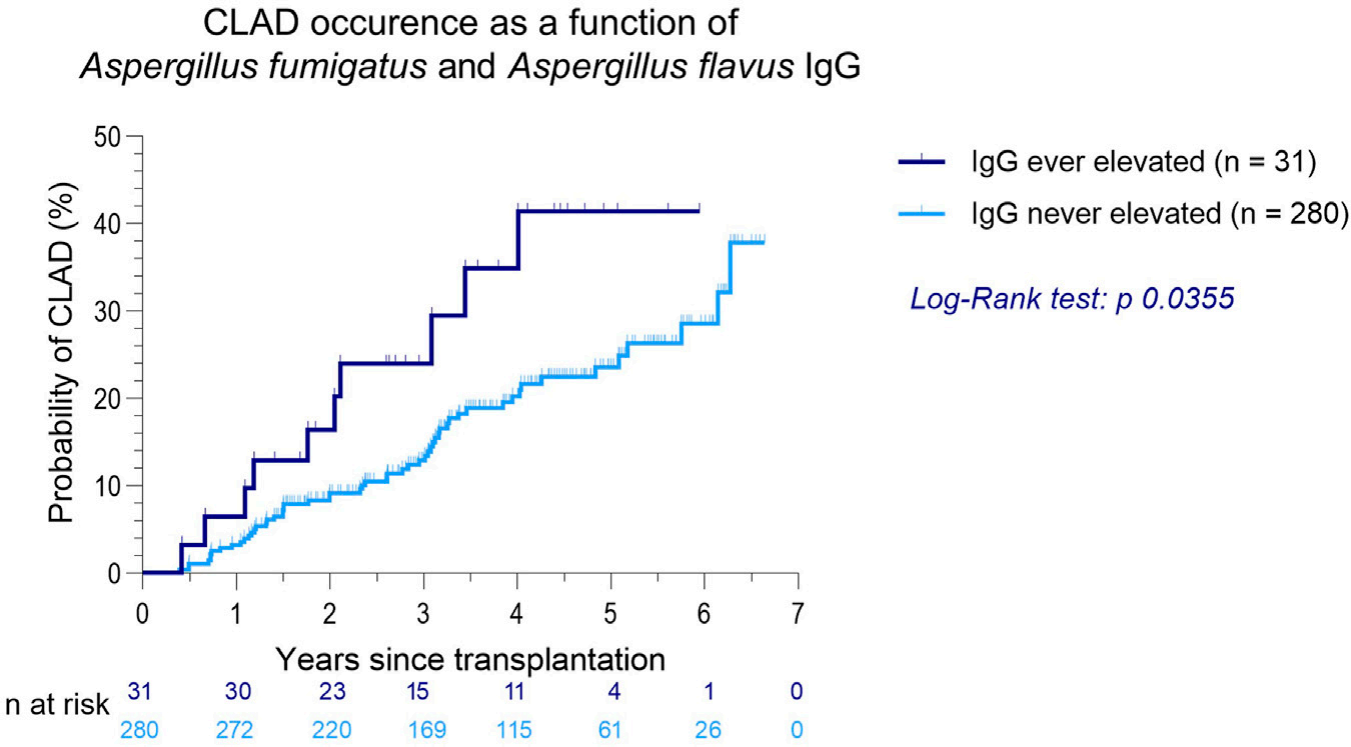
CLAD occurrence as a function of *Aspergillus fumigatus* and *Aspergillus flavus* IgG after LTx.

Elevated *Aspergillus fumigatus* or *flavus* IgG was associated with a significantly higher CLAD occurrence (*p* = 0.0355, Figure 2). AUC in receiver operating characteristic analysis was 0.58 (*p* = 0.0490). In patients with elevated *Aspergillus fumigatus* or *flavus* IgG, RAS as well as transition from BOS to RAS occurred more frequently (19.4% vs. 6.1%, Table 3).

Elevated *Aspergillus fumigatus, flavus* or *niger* IgE at 1, 2 or 3 years post-LTx was detected in 60/311 (19.3%) of patients, but was not associated with occurrence of *Aspergillus* species isolation in the consecutive or previous year, CLAD or death (data not shown).

### Fungal Isolation - CLAD/Survival

A total of 268 positive cultures for fungi were obtained in 47.6% of patients (*n* = 148), with a median of 1 [1–2] positive sample per patient. Time to first positive fungal airway culture was 326 days post-transplantation on average (note: survival to 1 year post-LTx was an inclusion criterium for this cohort and cultures during the first month post-LTx were excluded). Mold isolation after the first postoperative month was significantly associated with CLAD occurrence (*p* = 0.0011) and showed a predictive trend towards death (*p* = 0.0529).

Mold isolation after the first year post-LTx also remained associated with later CLAD occurrence (*p* = 0.0011). There was no relationship between positive donor mold cultures (*n* = 17) and recipient positive mold cultures (*n* = 12) during the first postoperative month (odds ratio 1.602, *p* = 0.49).

**FIGURE 3:**
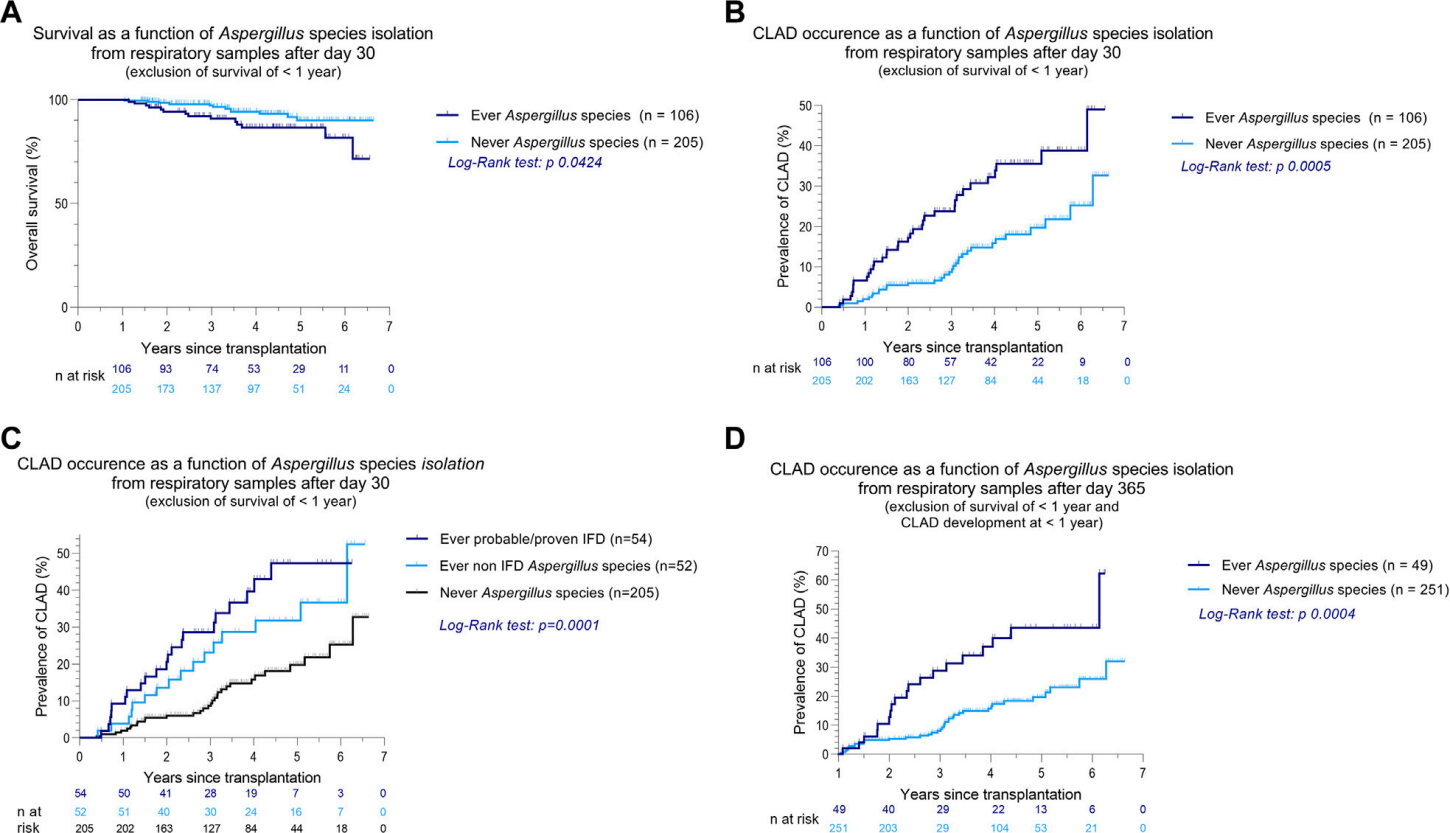
Kaplan-Meier curves of overall survival and CLAD occurrence as a function of *Aspergillus* species isolation from respiratory samples after LTx **(A–D)**. In all analyses, only patients with survival >1 year after LTx were included. **(C)** When a culture was positive for *Aspergillus* species, further clinical phenotyping was performed according to the latest EORTC-MSGERC and ISHLT consensus (15, 16). **(D)** Patients who developed CLAD in the first postoperative year were excluded. Abbreviations: LTx, lung transplantation; A., *Aspergillus*; CLAD, chronic lung allograft dysfunction.

*Aspergillus* species were cultured 110 times (74.3%), of which 92 were identified as *Aspergillus fumigatus* (62.1%), 11 as *Aspergillus flavus* (7.4%), and 4 as *Aspergillus niger* (2.7%). *Aspergillus* species isolation after the first postoperative month was significantly associated with both CLAD and death (*p* = 0.0005 and 0.0424, Figures 3A,B), and *Aspergillus* species isolation after the first year post-LTx remained associated with later CLAD occurrence (*p* = 0.0004, Figure 3D). In both IFD and non-IFD, *Aspergillus* species were associated with CLAD (*p* = 0.0001, Figure 3C) compared to patients in whom no *Aspergillus* species were isolated. Interestingly, there was no significant difference in CLAD occurrence between IFD and non-IFD *Aspergillus* isolation (*p* = 0.2970).

Most *Aspergillus* species were treated (66.0%), with either voriconazole, posaconazole, isavuconazole, or amphotericin B (54.8%, 34.4%, 6.5% and 4.3%, respectively). Distribution of the clinical phenotypes (stable, CLAD: BOS, RAS, BOS to RAS) in patients with or without fungal isolation is shown in Table 4.

**TABLE 4:** CLAD phenotype comparison in patients with or without fungal isolation from respiratory samples after LTx.

	Ever *Aspergillus* species (n = 106)	Never *Aspergillus* species (n = 205)	Ever molds (n = 136)	Never molds (n = 175)
BOS	20 (18.9%)	24 (11.7%)	24 (17.3%)	20 (11.4%)
RAS	13 (12.3%)	6 (2.9%)	14 (10.2%)	5 (2.9%)
BOS to RAS	3 (2.8%)	1 (0.5%)	3 (2.2%)	1 (0.6%)
Stable	70 (66.0%)	174 (84.9%)	95 (69.9%)	149 (85.1%)
	*p* = 0.0002		*p* = 0.0034	

## Discussion

This retrospective study of prospectively collected data in a large cohort of LTx recipients demonstrated for the first time that *Aspergillus* species specific IgG was associated with later fungal isolation and fungal-related complications (IFD,CLAD). This study also confirmed previously published findings that mold isolation, especially *Aspergillus* species, was associated with CLAD development and impaired survival (3–5).

CLAD is the leading cause of death beyond the first post-transplant year. Respiratory infections are among the many elements reported to contribute to CLAD (4, 5, 17–19). Indeed, presence of molds within the airways after transplantation may result in local innate inflammation and epithelial injury followed by dysregulation of repair mechanisms, ultimately responsible for chronic fibroproliferation and progressive graft dysfunction (4, 5, 20–22).

However, as mentioned previously, respiratory samples are not always available, as not all patients produce sputum and bronchoscopy is not always performed or possible. Subclinical presence of fungi could thus remain undetected. Fungal-specific IgG could therefore represent a new way of identifying lung transplant patients at risk for fungal-related complications, besides sampling of the airways.

*Aspergillus fumigatus* IgG levels were lower after LTx compared to pre-transplant, which is probably due to our immunosuppressive regimen as well as replacement of the diseased (and often, especially in CF patients, *Aspergillus* species colonized) native lungs with (non-colonized) donor lungs. *Aspergillus fumigatus* IgG levels pre- and post-LTx were correlated, which could possibly mean that these patients are inherently more prone to develop IgG (and thus have higher immune activity), or there is an ongoing environmental exposure in these patients. *Aspergillus* IgG levels were not significantly different at 1, 2 and 3 years post-LTx. *Aspergillus fumigatus* IgG was associated with *Aspergillus fumigatus* isolation in the previous or consecutive year, and patients with elevated *Aspergillus fumigatus* or *flavus* IgG has more mold and *Aspergillus* species isolation during the study period. This could be explained by the fact that specific IgG is a measure of fungal exposure, even when fungi are not always captured by routine follow-up respiratory samples, especially when frequency of follow-up decreases over time post-LTx. Fungus-specific IgG has been used in the diagnosis of (invasive) fungal infections such as chronic pulmonary aspergillosis and ABPA, but also in determining risk factors for interstitial lung diseases, such as hypersensitivity pneumonitis (6, 7, 23, 24). The clinical advantage of IgG measurement is that it is not dependent on sampling of the airways and can therefore always be measured. Elevated *Aspergillus fumigatus* or *flavus* IgG was associated with higher CLAD prevalence, which is not surprising, as fungal infections are a risk factor for CLAD. Interestingly, RAS as well as transition from BOS to RAS occurred more frequently in the group with elevated IgG compared to non-elevated IgG (Table 3), indicating that elevated IgG could be a marker for fungal-related complications predisposing more to RAS than BOS.

On the other hand, positive specific IgE for *Aspergillus fumigatus* was not significantly associated with CLAD, survival or fungal isolation in our cohort**.** This is probably due to the shorter half-life of IgE in the serum (only 2–3 days) and the fact that the majority of IgE in the body is cell-bound, and only a small fraction can be measured in the circulation. This is in contrast to IgG, which has a much longer half-life of about 3 weeks and is the most prevalent antibody molecule in the serum (25, 26).

Invasive fungal infections are a known risk factor for CLAD, and even without signs of infection *Aspergillus* species isolation from airway sampling is considered a risk factor for CLAD. In our study, *Aspergillus* species were cultured in 74.3% of the respiratory samples demonstrating molds, and *Aspergillus* species isolation was significantly associated with CLAD and impaired survival, confirming findings from earlier studies (2–5). There was no significant difference in CLAD occurrence in patients with *Aspergillus* IFD vs. non-IFD *Aspergillus* isolation. Median time from *Aspergillus* isolation (without signs of infection) to CLAD in earlier studies was reported to be 633 days (IQR 575-675) (20) or 261 days (21), while in our cohort, in the group that developed CLAD, the median time from *Aspergillus* species isolation (exclusion of first postoperative month) to CLAD diagnosis was 384 days, however *Aspergillus* species infections were also included in our cohort. Interestingly, patients with *Aspergillus* species isolation showed a higher tendency to develop RAS or transition from BOS to RAS phenotype (15.1% vs. 3.4% in patients without *Aspergillus* species isolation, Table 4). This may again indicate that fungal infections may predispose to RAS rather than to BOS, which requires validation in other cohorts.

Overall, elevated *Aspergillus*-specific IgG should therefore increase vigilance for *Aspergillus*-related complications.

There are some limitations to our study, such as its single center set-up, the low detection rate of elevated IgG levels and the lack of skin prick tests, as there is a potential discordance between *in vitro* tests and skin prick tests. However, the value of skin prick tests in LTx patients (who are on maintenance corticosteroids) is questionable. Also, only IgG specific for *Aspergillus fumigatus* and *flavus* was measured, and as these are the most prevalent *Aspergillus* species, other *Aspergillus* species were uncommon in respiratory cultures (only 4.8% of respiratory cultures). These IgGs thus covered the majority of isolated *Aspergillus* species. Also, there is a lack of assessment of local immune activation (i.e., BAL or blood lymphocytic subtypes) or immunosuppressive treatment, which were outside the scope of the study.

In conclusion, fungus-specific IgG could be a useful non-invasive marker of fungal exposure in long-term follow up after LTx, to help identify patients at risk for fungal-related complications, CLAD, and inferior outcome.

## Data Availability

The raw data supporting the conclusion of this article will be made available by the authors, without undue reservation.

## References

[1] Nosotti M Tarsia P Morlacchi LC. Infections after Lung Transplantation. J Thorac Dis (2018) 10:3849–68. 10.21037/jtd.2018.05.204 30069386 PMC6051843

[2] Pasupneti S Manouvakhova O Nicolls MR Hsu JL. Aspergillus-related Pulmonary Diseases in Lung Transplantation. Med Mycol (2017) 55:96–102. 10.1093/mmy/myw121 27816902 PMC6388974

[3] Meyer KC Raghu G Verleden GM Corris PA Aurora P Wilson KC An International ISHLT/ATS/ERS Clinical Practice Guideline: Diagnosis and Management of Bronchiolitis Obliterans Syndrome. Eur Respir J (2014) 44:1479–503. 10.1183/09031936.00107514 25359357

[4] Weigt SS Finlen Copeland CA Derhovanessian A Shino MY Davis WA Snyder LD Colonization with Small Conidia Aspergillus Species Is Associated with Bronchiolitis Obliterans Syndrome: A Two-Center Validation Study. Am J Transplant (2013) 13:919–27. 10.1111/ajt.12131 23398785 PMC3618528

[5] Le Pavec J Pradère P Gigandon A Dauriat G Dureault A Aguilar C Risk of Lung Allograft Dysfunction Associated with Aspergillus Infection. Transpl Direct (2021) 7:e675. 10.1097/TXD.0000000000001128 PMC8184025 34113715

[6] Patterson TF Thompson GR Denning DW Fishman JA Hadley S Herbrecht R Practice Guidelines for the Diagnosis and Management of Aspergillosis: 2016 Update by the Infectious Diseases Society of America. Clin Infect Dis (2016) 63:e1–60. 10.1093/cid/ciw326 27365388 PMC4967602

[7] Denning DW Cadranel J Beigelman-Aubry C Ader F Chakrabarti A Blot S Chronic Pulmonary Aspergillosis: Rationale and Clinical Guidelines for Diagnosis and Management. Eur Respir J (2016) 47:45–68. 10.1183/13993003.00583-2015 26699723

[8] Kao CC Hanania NA Parulekar AD. The Impact of Fungal Allergic Sensitization on Asthma. Curr Opin Pulm Med (2021) 27:3–8. 10.1097/MCP.0000000000000740 33027187

[9] Chen H Zhang X Zhu L An N Jiang Q Yang Y Clinical and Immunological Characteristics of Aspergillus Fumigatus-Sensitized Asthma and Allergic Bronchopulmonary Aspergillosis. Front Immunol (2022) 13:939127. 10.3389/fimmu.2022.939127 35983066 PMC9379317

[10] Singer A Ali FR Quantrill S North N Stevens M Lambourne J Utility of Immunology, Microbiology, and Helminth Investigations in Clinical Assessment of Severe Asthma. J Asthma (2022) 59:541–51. 10.1080/02770903.2020.1868496 33356678

[11] Hedayati N Mortezaee V Mahdaviani SA Mirenayat MS Hassanzad M Pourabdollah M Prevalence of Specific Immunoglobulin E and G against Aspergillus fumigatus in Patients with Asthma. Curr Med Mycol (2018) 4:7–11. 10.18502/cmm.4.4.380 PMC6386509 30815611

[12] Everaerts S Lagrou K Dubbeldam A Lorent N Vermeersch K Van Hoeyveld E Sensitization to Aspergillus fumigatus as a Risk Factor for Bronchiectasis in COPD. Int J Chron Obstruct Pulmon Dis (2017) 12:2629–38. 10.2147/COPD.S141695 28919731 PMC5587018

[13] Alghamdi NS Barton R Wilcox M Peckham D. Serum IgE and IgG Reactivity to Aspergillus Recombinant Antigens in Patients with Cystic Fibrosis. J Med Microbiol (2019) 68:924–9. 10.1099/jmm.0.000991 31090534

[14] Verleden GM Glanville AR Lease ED Fisher AJ Calabrese F Corris PA Chronic Lung Allograft Dysfunction: Definition, Diagnostic Criteria, and Approaches to treatment―A Consensus Report from the Pulmonary Council of the ISHLT. J Heart Lung Transplant (2019) 38:493–503. 10.1016/j.healun.2019.03.009 30962148

[15] Donnelly JP Chen SC Kauffman CA Steinbach WJ Baddley JW Verweij PE Revision and Update of the Consensus Definitions of Invasive Fungal Disease from the European Organization for Research and Treatment of Cancer and the Mycoses Study Group Education and Research Consortium. Clin Infect Dis (2020) 71:1367–76. 10.1093/cid/ciz1008 31802125 PMC7486838

[16] Husain S Mooney ML Danziger-Isakov L Mattner F Singh N Avery R A 2010 Working Formulation for the Standardization of Definitions of Infections in Cardiothoracic Transplant Recipients. J Heart Lung Transpl (2011) 30:361–74. 10.1016/j.healun.2011.01.701 PMC7172457 21419994

[17] Verleden SE Ruttens D Vandermeulen E Vaneylen A Dupont LJ Van Raemdonck DE Bronchiolitis Obliterans Syndrome and Restrictive Allograft Syndrome: Do Risk Factors Differ? Transplantation (2013) 95:1167–72. 10.1097/TP.0b013e318286e076 23425818

[18] De Muynck B Van Herck A Sacreas A Heigl T Kaes J Vanstapel A Successful *Pseudomonas aeruginosa* Eradication Improves Outcomes after Lung Transplantation: a Retrospective Cohort Analysis. Eur Respir J (2020) 56:2001720. 10.1183/13993003.01720-2020 32471935

[19] Moore CA Pilewski JM Venkataramanan R Robinson KM Morrell MR Wisniewski SR Effect of Aerosolized Antipseudomonals on Pseudomonas Positivity and Bronchiolitis Obliterans Syndrome after Lung Transplantation. Transpl Infect Dis (2017) 19:e12688. 10.1111/tid.12688 28273385

[20] Weigt SS Wang X Palchevskiy V Patel N Derhovanessian A Shino MY Gene Expression Profiling of Bronchoalveolar Lavage Cells during Aspergillus Colonization of the Lung Allograft. Transplantation (2018) 102:986–93. 10.1097/TP.0000000000002058 29256975 PMC5962368

[21] Weigt SS Elashoff RM Huang C ArdehAli A Gregson AL KuBak B Aspergillus Colonization of the Lung Allograft Is a Risk Factor for Bronchiolitis Obliterans Syndrome. Am J Transpl (2009) 9:1903–11. 10.1111/j.1600-6143.2009.02635.x PMC4214373 19459819

[22] Gregson AL Wang X Weigt SS Palchevskiy V Lynch JP Ross DJ Interaction between Pseudomonas and CXC Chemokines Increases Risk of Bronchiolitis Obliterans Syndrome and Death in Lung Transplantation. Am J Respir Crit Care Med (2013) 187:518–26. 10.1164/rccm.201207-1228OC 23328531 PMC3733405

[23] Richardson M Page I. Role of Serological Tests in the Diagnosis of Mold Infections. Curr Fungal Infect Rep (2018) 12:127–36. 10.1007/s12281-018-0321-1 30294405 PMC6153857

[24] Samson MH Vestergaard JM Knudsen CS Kolstad HA. Serum Levels of IgG Antibodies against *Aspergillus fumigatus* and the Risk of Hypersensitivity Pneumonitis and Other Interstitial Lung Diseases. Scand J Clin Lab Invest (2021) 81:451–3. 10.1080/00365513.2021.1943758 34278893

[25] Lawrence MG Woodfolk JA Schuyler AJ Stillman LC Chapman MD Platts-Mills TAE. Half-life of IgE in Serum and Skin: Consequences for Anti-IgE Therapy in Patients with Allergic Disease. J Allergy Clin Immunol (2017) 139:422–8. 10.1016/j.jaci.2016.04.056 27496596 PMC5405770

[26] Charles AJ Travers P Walport M Shlomchik MJ. Allergy and Hypersensitivity. In: Immunobiology: The Immune System in Health and Disease 5th Edition (2001). Available from: https://www.ncbi.nlm.nih.gov/books/NBK10756/ (accessed June 2, 2022).

